# VMAT–SBRT planning based on an average intensity projection for lung tumors located in close proximity to the diaphragm: a phantom and clinical validity study

**DOI:** 10.1093/jrr/rrv058

**Published:** 2015-09-28

**Authors:** Shingo Ohira, Yoshihiro Ueda, Misaki Hashimoto, Masayoshi Miyazaki, Masaru Isono, Hiroshi Kamikaseda, Akira Masaoka, Masaaki Takashina, Masahiko Koizumi, Teruki Teshima

**Affiliations:** 1 Department of Radiation Oncology, Osaka Medical Center for Cancer and Cardiovascular Diseases, Nakamichi 1-3-3, Higashinari-ku, Osaka, 537–8511, Japan; 2 Department of Medical Physics and Engineering, Osaka University Graduate School of Medicine, Suita, Japan; 3 Department of Radiation Oncology, Osaka University Graduate School of Medicine, Suita, Japan

**Keywords:** AIP, VMAT, SBRT, lung, diaphragm

## Abstract

The aim of the this study was to validate the use of an average intensity projection (AIP) for volumetric-modulated arc therapy for stereotactic body radiation therapy (VMAT–SBRT) planning for a moving lung tumor located near the diaphragm. VMAT–SBRT plans were created using AIPs reconstructed from 10 phases of 4DCT images that were acquired with a target phantom moving with amplitudes of 5, 10, 20 and 30 mm. To generate a 4D dose distribution, the static dose for each phase was recalculated and the doses were accumulated by using the phantom position known for each phase. For 10 patients with lung tumors, a deformable registration was used to generate 4D dose distributions. Doses to the target volume obtained from the AIP plan and the 4D plan were compared, as were the doses obtained from each plan to the organs at risk (OARs). In both phantom and clinical study, dose discrepancies for all parameters of the dose volume (D_min_, D_99_, D_max_, D_1_ and D_mean_) to the target were <3%. The discrepancies of D_max_ for spinal cord, esophagus and heart were <1 Gy, and the discrepancy of V20 for lung tissue was <1%. However, for OARs with large respiratory motion, the discrepancy of the D_max_ was as much as 9.6 Gy for liver and 5.7 Gy for stomach. Thus, AIP is clinically acceptable as a planning CT image for predicting 4D dose, but doses to the OARs with large respiratory motion were underestimated with the AIP approach.

## INTRODUCTION

The aim of stereotactic body radiation therapy (SBRT) is to deliver sufficient doses to control a tumor with a small number of fractions while minimizing the exposure to surrounding organs at risk (OARs). For lung cancer, promising outcomes with excellent control rates have been reported, and a higher dose to the tumor seems to have yielded better local control [1, 2]. Recently, an ablative type of SBRT that is designed to generate an inhomogeneous dose distribution for a given target volume has become popular. In Radiation Therapy Oncology Group (RTOG) protocol 0618, the maximum dose within the target volume was specified as 15–40% higher than the prescribed dose [3]. For such treatment, volumetric-modulated arc therapy (VMAT) has the advantages of fast dose delivery, high dose conformity to the target volume, and sparing of OARs [4, 5]. However, certain characteristic features of VMAT, such as continuous variations in multileaf collimator (MLC) positions and gantry speed, have raised concerns about using VMAT for tumors moving continuously with respiration.

One such concern is whether the effect of interplay between tumor motion and MLC motions will result in an underdose or overdose in relation to the target volume. Jiang *et al.* reported that the dose discrepancy due to the interplay effect was <2% for 30 fractions in a static intensity-modulated radiation field, although the effect caused a dose discrepancy of up to 18% in a single fraction [6]. For VMAT–SBRT, several investigators have shown that there are limited impacts of the interplay effect on target dose because of delivering a few thousand monitor units [7–9]. Therefore, the interplay effect on the target dose may not be significant.

A remaining concern is how an accurate treatment plan can be generated for a moving target. Four-dimensional computed tomography (4DCT) is a standard modality for assessing patient motion. Chin *et al.* introduced a 4D VMAT treatment planning technique that incorporates 4DCT directly into an optimization process [10]. This method resulted in accurate dose calculations for the moving target and high dose sparing of surrounding OARs. It is essential that patient motion at simulation is synchronized with that at treatment, but due to the complicated process involved in such synchronization, this is not yet ready for clinical use. A clinical treatment plan using the VMAT technique was optimized on the basis of only one CT image set. A common approach for treatment planning is the use of an average intensity projection (AIP), which is generated by averaging pixel densities for all phase images of 4DCT. Previous studies demonstrated the use of AIP was an effective strategy for designing treatment plans for patients with lung cancer [11–13]. Although each study included several clinical cases, it should be remembered that a tumor can develop anywhere on a lung tissue, and its characteristics can vary widely in size, motion range, location, etc.

We have occasionally encountered a tumor that was located in close proximity to the diaphragm. Because of the respiratory motion of the tumor and the diaphragm in cases such as these, their shapes on the AIP are blurred, with the extent depending on the respiratory form of each patient. When treatment planning is based on such a blurred AIP, the optimization process leads to concern about whether the fluence is greater or less than the ideal fluence for delivery of a sufficient dose to the target on the AIP. Consequently, the actual dose may differ from the expected dose on the treatment plan. It therefore remains controversial whether treatment planning based on the AIP can still be used for such a moving target and the OARs. The aim of the phantom and clinical studies presented here was to check the validity of AIP-based VMAT–SBRT treatment planning for lung tumors located in close proximity to the diaphragm.

## MATERIALS AND METHODS

### Phantom study

#### Acquisition of 4DCT

**Fig. 1.**
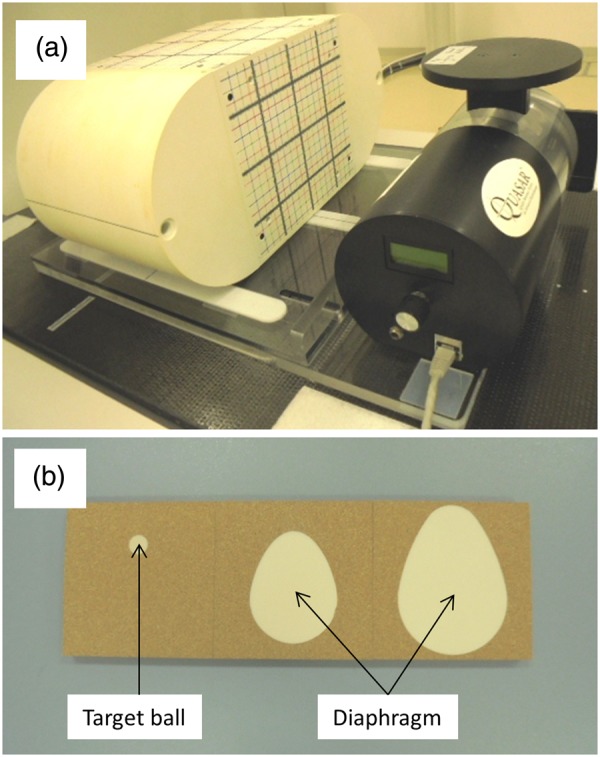
(**a**) A dynamic phantom consisted of I'mRT phantom and Quasar motion platform. (**b**) A lung cubic insert consisted of cork plates in which a 20-mm target ball and a diaphragm dome were embedded.

**Fig. 2.**
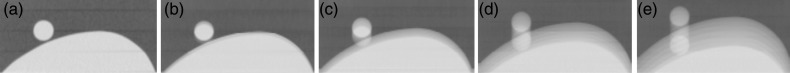
Sagittal views of the dynamic phantom with the target ball located near the diaphragm: (**a**) static and AIPs with images for (**b**) 5-, (**c**) 10-, (**d**) 20- and (**e**) 30-mm motion.

A dynamic phantom previously introduced by us was used for this study (Fig. 1a) [14]. The phantom comprised a body phantom with cubic lung insert (I'mRT Phantom; IBA Dosimetry, Schwarzenbruck, Germany), and a programmable motion platform (Quasar Respiratory Motion Platform; Modus Medical Devices Inc., London, ONT, Canada). In this study, the cubic lung insert consisted of cork plates in which a 20-mm target ball and a diaphragm dome made from water-equivalent material were closely embedded (Fig. 1b). CT images were acquired using a CT simulator (LightSpeed 16; GE Medical Systems, Waukesha, WI), which was equipped with the Real-time Position Management (RPM) system (Varian Medical Systems, Palo Alto, CA). The parameters for acquisition were 1.25-mm slice thickness, 512 × 512 matrix, and 50 × 50 cm field of view (FOV). First, the static phantom image was acquired at the center of phantom motion, as shown in Fig. 2a. Thereafter, 4DCT images were acquired, with the motion platform programmed for biquadratic sinusoidal profiles so that the time the target ball spent on the superior side to the center of motion was longer. The motion amplitudes were 5, 10, 20 and 30 mm, with a 6-s period for all amplitudes. After acquisition, the images were transferred to a workstation (Advantage Sim, GE Medical Systems), which generated 10 respiratory bins on phase-based sorting. AIPs were generated from all these bins (Fig. 2b–e).

#### Treatment planning

The target ball on the static image was contoured to represent the gross tumor volume (GTV) by using a treatment planning system (TPS) (Eclipse, version 11.0, Varian Medical Systems). The internal target volume (ITV) was generated to include the volume of the target ball at every phase. A planning target volume (PTV) was generated with a 3D margin of 5 mm around the ITV, and VMAT–SBRT plans were created based on each AIP. All doses for planning in this study (phantom and clinical) were calculated by means of an analytic anisotropic algorithm with heterogeneity correction. The treatment plans were designed to stipulate 48 Gy in four fractions, so as to cover 95% of the PTV volumes by using four half-gantry arcs, while the maximum dose within the ITV was specified as not less than 115% or more than 145% of the prescribed dose. The treatment plans were copied into the static phantom image, together with changes in isocenter positions, so that the known biquadratic sinusoidal phantom motion at each phase was reproduced in the TPS. Except for the isocenter position, all planning parameters (beam arrangement, leaf positions, number of monitor units etc.) remained consistent. Thereafter, doses were recalculated based on the static phantom. The 4D dose distribution that represented the actual dose delivered to the phantom was obtained by accumulating the dose distributions.

The discrepancies of the doses to the target ball in the AIP and in the 4D plan were determined by subtracting the dose–volume parameters of the ITV in the AIP plan from those of the GTV in the 4D plan. The dose discrepancies were expressed as a percentage by dividing these discrepancies obtained with subtraction by the corresponding the dose–volume parameters of the 4D plan.

### Clinical study

#### Treatment simulation and planning

Table 1 lists characteristics of the 10 patients enrolled in this study. The criterion for selection was that part of the tumor in the inhale phase was overlapped by the diaphragm in the exhale phase. The volume of the ITV overlapped by the diaphragm was then measured, and the tumor motion was measured by the distance between the center of the tumor on the exhale phase and the center of the tumor on the inhale phase. For the simulation, all patients were immobilized with the BodyFix double-vacuum immobilization system (Medical Intelligence, Schwabmuenchen, Germany). 4DCT was administered with the patients under quiet respiration. Acquisition parameters were 2.5-mm slice thickness, 512 × 512 matrix, and 50 × 50 cm FOV. The AIP images were then created from the 4DCT images in the same manner as in the phantom study.

**Table 1.** Patient characteristics

Patient #	Location	GTV, cm^3^	ITV, cm^3^	Overlap, cm^3^	Motion, cm
1	Right	4.3	13.2	2.9	1.7
2	Right	24.9	41.3	15.1	2.7
3	Right	21.6	39.6	0.9	2.3
4	Right	4.6	8.2	0.5	1.0
5	Left	7.0	20.0	6.9	3.2
6	Left	8.4	20.2	6.4	2.9
7	Right	2.0	4.8	0.7	0.8
8	Right	4.0	8.3	2.8	1.1
9	Left	0.8	3.6	1.6	2.4
10	Right	1.5	6.9	3.0	2.0

**Fig. 3.**
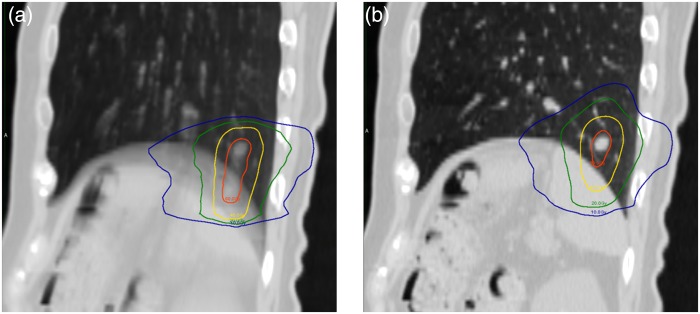
Sagittal views of the (**a**) AIP and (**b**) 4D treatment plans for Patient 9. Blue, green, yellow and red lines indicate the isodose lines of 10, 20, 40 and 60 Gy, respectively.

GTV was determined by a radiation oncologist by using the tumor on the full exhale phase image of the 4D-CT (ex-CT). The definitions of ITV and PTV were the same as in the phantom study. OARs were delineated on both the AIP and ex-CT to determine doses for OARs in the AIP and the 4D plans, so that the volumes of the OARs differed substantially between these two datasets due to the motion artifact of the organs. A treatment plan was generated based on the AIP by using three to five half-gantry arcs (Fig. 3a). The prescribed dose for the PTV was the same as in the phantom study. Dose constraints for OARs were: D_max_ for the spinal cord, bowel, stomach, esophagus and heart were 20, 16, 25, 25 and 40 Gy, respectively; percentage of lung volume exceeding 20 Gy (V20) was <10%.

#### 4D dose calculation

The treatment plan from the AIP was copied into each phase image of 4DCT with all planning parameters remaining consistent. Subsequently, the doses were recalculated based on each 4DCT phase image. For the clinical study, a deformable image registration (DIR) was required to create the 4D dose distribution. As the ex-CT was a reference image, each phase image had to be deformed to generate a deformation map. A visual inspection was performed for this procedure to ensure the deformations of the tumor and the OARs were properly carried out. Each dose distribution was accumulated into the reference image by using the corresponding deformation maps (Fig. 3b).

As with the phantom study, the dose discrepancy between the target in the AIP and the 4D plan was calculated. In addition to determination of the target dose, the dose discrepancies in the surrounding OARs (spinal cord, esophagus, heart, liver, bowel, stomach and lung) were also calculated by subtracting doses in the AIP plan from those in the 4D plan. For cases with tumors located in the right lung, the dose to the liver was assessed; on the other hand, when tumors were located in the left lung, the doses to the bowel and stomach were assessed. For lung tissue, the contoured volume not including the PTV was evaluated. The differences in dose–volume parameters between the AIP and the 4D plans were analyzed by means Wilcoxon's signed rank test (SPSS, version 16; SPSS Inc., Chicago, IL).

## RESULTS

### Phantom study

Table 2 shows the discrepancies between the dose–volume parameters (D_min_, D_99_, D_max_, D_1_ and D_mean_) for the target ball in the phantom study using the AIP and the 4D plan. The dose discrepancies were positive under all experimental conditions, indicating that the accumulated 4D dose was higher than the planned dose on the AIP. Overall, the larger the phantom motion, the larger the dose discrepancy, with D_1_ for 3-cm phantom motion showing the largest discrepancy of 2.0 Gy (3.0%).

**Table 2.** Dose discrepancy of target ball for phantom study

	Discrepancy, Gy (%)				
Motion, cm	D_min_	D_99_	D_max_	D_1_	D_mean_
0.5	0.1 (0.2)	0.5 (0.8)	0.1 (0.1)	0.2 (0.2)	0.9 (1.4)
1.0	0.1 (0.2)	0.4 (0.7)	0.5 (0.8)	0.5 (0.8)	1.0 (1.5)
2.0	0.6 (1.1)	0.7 (1.2)	1.5 (2.3)	1.8 (2.6)	1.5 (2.5)
3.0	0.6 (1.1)	0.6 (1.1)	1.6 (2.5)	2.0 (3.0)	1.7 (2.7)

D_min_ = minimum dose, D_99_ = dose to 99% of the volume, D_max_ = maximum dose, D_1_ = dose to 1% of the volume, D_mean_ = mean dose.

### Clinical study

The average ± standard deviation (SD) for D_min_, D_99_, D_max_, D_1_ and D_mean_ of the AIP plan for 10 patients were 55.1 ± 1.7, 56.9 ± 1.3, 66.7 ± 2.1, 65.9 ± 2.1 and 61.7 ± 1.6 Gy, respectively, and the corresponding values for the 4D plan were 55.2 ± 1.6, 57.0 ± 1.5, 66.4 ± 2.5, 66.0 ± 2.5 and 62.2 ± 1.6 Gy. Only D_mean_ showed a significant difference (*P* < 0.01). Table 3 shows the dose discrepancies between the AIP and the 4D plan for each patient. The largest discrepancy was 1.5 Gy (2.7%) for D_min_ for Patient 2, but in most of the cases the dose discrepancies were within 2%.

**Table 3.** Dose discrepancy of tumors for clinical study

Discrepancy, Gy (%)					
Patient #	D_min_	D_99_	D_max_	D_1_	D_mean_
1	−0.1 (–0.2)	−0.2 (–0.3)	−1.5 (–2.4)	−0.4 (–0.6)	0.5 (0.8)
2	1.5 (2.7)	0.9 (1.6)	−1.4 (–2.1)	−0.9 (–1.4)	0.8 (1.3)
3	−0.6 (–1.1)	−0.3 (–0.5)	−0.4 (–0.6)	0.0 (0.0)	0.6 (0.9)
4	0.2 (0.3)	0.1 (0.3)	−0.4 (–0.5)	0.0 (0.0)	0.6 (1.0)
5	−0.1 (–0.1)	−0.3 (–0.6)	−0.1 (–0.1)	0.1 (0.2)	0.2 (0.3)
6	−0.4 (–0.7)	−0.3 (–0.4)	0.0 (0.0)	0.4 (0.5)	0.8 (1.4)
7	0.4 (0.6)	0.6 (1.1)	1.1 (1.8)	1.2 (1.6)	1.0 (1.6)
8	0.1 (0.2)	−0.3 (–0.6)	−0.8 (–1.2)	−0.5 (–0.8)	0.2 (0.3)
9	−1.3 (–2.3)	−0.7 (–1.2)	−0.2 (–0.3)	0.2 (0.3)	0.1 (0.1)
10	1.4 (2.5)	1.4 (2.4)	0.6 (0.9)	1.0 (1.4)	0.5 (0.8)

For spinal cord, esophagus, heart, liver, bowel and stomach, the average ± SD of the D_max_ for AIP plans were 8.8 ± 4.2, 7.4 ± 2.2, 15.4 ± 10.9, 56.0 ± 10.7, 12.5 ± 2.3 and 14.7 ± 6.1 Gy, respectively, and for 4D plans they were 8.8 ± 4.2, 7.4 ± 2.1, 15.1 ± 10.6, 58.7 ± 7.5, 13.3 ± 2.6 and 18.0 ± 4.2 Gy, respectively. The average ± SD of the V20 and D_mean_ of AIP plans to the lung tissue were 3.5 ± 1.7% and 2.9 ± 0.9 Gy, and of 4D plans they were 3.1 ± 1.8% and 2.8 ± 0.9 Gy, respectively. The values of both parameters of AIP plans to the lung tissue were significantly higher than those of the 4D plan (*P* < 0.05). Table 4 shows the discrepancies in doses to the OARs between the AIP and the 4D plan for each patient. For OARs with minor respiratory motion (spinal cord, esophagus and heart), discrepancies in D_max_ between AIP and 4D plans were within 1 Gy; for lung tissue, the discrepancies in V20 and D_mean_ for all patients were within 1% and 1 Gy, respectively. However, for the liver, bowel and stomach, which feature large respiratory motion, doses were underestimated more often with the AIP plan than with the 4D plan in most cases, and the dose discrepancy was as much as 9.6 Gy for the liver of Patient 3 and 5.7 Gy for the stomach of Patient 6.

**Table 4.** Dose discrepancy of OARs for clinical study

	Discrepancy, Gy (%)						
Patient #	Spinal cord	Esophagus	Heart	Lung	Liver	Bowel	Stomach
	D_max_	D_max_	D_max_	V20 (%)	D_max_	D_max_	D_max_
1	0.0	0.1	0.3	−0.3	3.0		
2	0.2	0.3	−0.1	−0.1	3.2		
3	−0.6	0.0	−0.4	0.0	9.6		
4	0.0	0.3	0.0	−0.2	0.5		
5	0.0	0.1	0.2	−1.0		1.0	0.9
6	−0.2	−0.8	−0.5	−0.6		0.9	5.7
7	0.0	0.0	−0.9	−0.6	3.4		
8	0.4	0.2	0.6	−0.2	−0.7		
9	0.0	−0.2	−0.4	−0.6		0.5	3.3
10	0.0	−0.5	−0.3	−0.5	0.0		

## DISCUSSION

The AIP-based VMAT–SBRT plans focusing on a moving target located near the diaphragm were validated. In both a phantom study and a clinical study of 10 patients, the AIP approach proved to be an effective and practical method in terms of doses delivered to the targets. However, doses to OARs with large respiratory motion, such as liver and stomach, were underestimated in the AIP plans.

The aim of the phantom experiment was to evaluate the effects of target and diaphragm motion on 4D dose distribution, while avoiding the uncertainty of the dose accumulations when using DIR, because the inaccuracy of DIR may have a significant dosimetric impact on cumulative dose distributions [15]. Glide-Hurst *et al.* used numerical phantoms, in which the target ball and the diaphragm were embedded in the lung medium, to validate the use of AIP for conventional–SBRT planning [13]. In their experiments, in cases where the phantom motion varied from 2 to 4 cm, the dose discrepancy between the planned and 4D cumulative dose to the target (D_min_, D_99_, D_mean_ and D_1_) was within 2%. Similarly to the results reported by Glide-Hurst *et al.*, dose discrepancies in our phantom experiments were limited. However, all dose–volume parameters of the 4D plan in our study were higher than those of the AIP plan, and the discrepancy increased with the magnitude of the phantom motion. This phenomenon can be explained by the fact that when the phantom motion was large, the shapes of the target and the diaphragm on the AIP became severely blurred, and the CT number as part of an ITV decreased. Optimization applied to such an AIP was thought to result in stronger fluence than the ideal due to lack of scatter contribution.

Ehlera *et al.* compared IMRT plans based on AIP with 4D plans generated by using DIR for eight patients with lung cancer. They reported that optimization for the AIP resulted in a uniform GTV dose throughout the breathing cycle, and none of the cases resulted in an equivalent uniform dose of less than the prescribed dose [12]. Admiraal *et al.* showed that when the target dose is set for the ITV and dose calculations are performed for the AIP, the 4D dose to the target compares favorably with the planned dose to the ITV [16]. The findings of these studies indicate that AIP-based treatment planning does not seem to make a significant difference in the dosimetric error. However, the typical tumor motion examined in these studies was within 2 cm. Glide-Hurst *et al.* evaluated the AIP-based treatment planning for conventional lung SBRT, and the worst-case scenario for the four cases investigated was of the tumor abutting the diaphragm showing a motion of 2 cm [13]. In our study, all tumors were located in close proximity to the diaphragm, and six of them moved >2 cm. These tumors were considered to be the least suitable for the use of AIP. It is undesirable for the actual dose distribution to provide less target coverage than the planned dose distribution and to cause a cold dose spot inside the whole target volume. Tomé *et al.* reported that serious reduction in the tumor control probability might occur if 1% of the target volume received <20% of the prescription dose [17]. Among the cases in our study where the planned dose was less than the actual dose, the largest dose discrepancy of the D_min_ and D_99_ to the target was –2.3 and –1.2%, respectively. This minor dose discrepancy seems to be clinically insignificant based on the fact that the cold spot caused inside the target volume was <3% of the planned dose, and this lends support to the conclusion that AIP-based treatment planning is a clinically acceptable approach for estimating the actual dose for lung tumors, even when they are in close proximity to the diaphragm, with its relatively large motion.

For OARs with small respiratory motion (spinal cord, esophagus and heart), dose discrepancies between AIP and 4D plans were negligible, but for those with large respiratory motion, dose discrepancies were prominent. The planned dose to the lung tissue was significantly higher than the actual dose. Because the treatment plan for AIP was designed to deliver a sufficient dose to the PTV, which was generated from the ITV with an additional margin, the volume of the lung on the AIP plan scheduled for high-dose irradiation was larger than that on the 4D plan. However, the dose discrepancy was limited and appeared to be clinically insignificant. For the liver, bowel and stomach, the doses estimated with the AIP plan were less than those estimated with the 4D plan. These findings could be explained by the fact that OARs included in the irradiated volume at the exhale phase were greater in volume than those at the inhale phase. Due to the blurred image quality, the shape of the organs contoured on the AIP was more like that at the inhale phase than at the exhale phase. Because the liver is considered to be a parallel organ, the dose discrepancy may not be clinically significant. However, for organs with lower tolerance for irradiation, such as bowel and stomach, underestimation of the dose on the AIP plan could cause serious harm in large-dose hypofractionated treatment. Therefore, we consider 4D dose calculation to be essential for predicting the actual dose to such organs. Otherwise, treatment with larger fraction number, e.g. 60 Gy in 10 fractions, might be required to reduce the biological effect of radiation for OARs.

A new radiotherapy modality of respiratory-gated VMAT could offer a solution for predicting actual doses to the targets as well as to the OARs. Gating irradiates the tumor only when it is in a given location, that is when it is near the exhale phase with a duty cycle of 25–70% [18]. Therefore, treatment planning for a gated VMAT does not require the AIP generated from full-phase image sets of 4DCT, and the optimization process can be carried out without being affected by the lack of scatter contribution. Moreover, this method would diminish the interplay effect on a target dose. The resultant planned dose distribution would constitute an accurate representation of the actual dose to a target and OARs. However, gated VMAT is only available for a limited number of machines due to the complexity resulting from repeated interruptions by the gating signal during VMAT dose delivery. Real-time target tracking with CyberKnife would be another solution for a moving target. Chan *et al.* evaluated 4D dose distributions of CyberKnife and VMAT in lung SBRT and concluded that CyberKnife had some advantage over free-breathing VMAT in the treatment of tumors showing large motion range and/or which are surrounded by multiple critical organs [19].

Recently, another approach for lung VMAT–SBRT planning has been proposed by Wiant *et al.* [20]. In their phantom study, treatment plans based on free-breathing image sets with the ITV set at tumor density and the PTV minus ITV set to a density intermediate between lung and tumor resulted in a reduction in beam modulation and significantly higher gamma passing rates than the ones based on AIP. Moreover, in their clinical study of five patients, the tumor volume was covered by the prescription dose for all respiratory phases, and the normal lung irradiation was reduced. That method might thus be a more effective approach than AIP for predicting the actual dose.

In conclusion, the phantom and clinical study presented here demonstrated the feasibility of AIP-based VMAT–SBRT planning. The AIP approach was found to be practical, and the dose discrepancies between the AIP and 4D plans were clinically acceptable (<3%). AIP could therefore constitute an approach to be recommended for a moving target located in close proximity to a diaphragm. However, the AIP approach underestimated doses for OARs with large respiratory motion. We thus consider 4D dose calculation to be essential when such OARs are located near the irradiated target.

## FUNDING

Funding to pay the Open Access publication charges for this article was provided by a grant from the Japan Society for the Promotion of Science (JSPS) Core-to-Core Program (Grant No. 23003).
